# Tackling the increasing contamination of the water supply by iodinated contrast media

**DOI:** 10.1186/s13244-022-01175-x

**Published:** 2022-02-24

**Authors:** Helena M. Dekker, Gerard J. Stroomberg, Mathias Prokop

**Affiliations:** 1 Department of Medical Imaging, Radboud University Medical Center, Geert Grooteplein Zuid 10, 6525 GA Nijmegen, The Netherlands; 2 RIWA-Rijn – Association of River Water Works, Groenendael 6, 3439 LV Nieuwegein, The Netherlands

**Keywords:** Contrast media, Environmental fate, Water supply system, Sustainability

## Abstract

Contrast media are essential for diagnostic and interventional procedures. Iodinated contrast media are the most commonly used agents, with CT requiring the largest overall quantities. Data show that these iodinated contrast media are found in sewage water, surface water and drinking water in many regions in the world. Because standard drinking water purification techniques only provide poor to moderate removal of iodinated contrast media, these substances pose a problem for drinking water preparation that has not yet been solved. There is a growing body of evidence supporting the negative environmental effects of iodinated contrast media via their breakdown products. The environmental impact of iodinated contrast media can be mitigated by measures focusing on the application of contrast media or the excretion of contrast media. Measures with respect to contrast application include reducing the utilization of contrast media, reducing the waste of contrast media and collecting residues of contrast media at the point of application. The amount of contrast media excreted into the sewage water can be decreased by introducing urine bags and/or special urine collection and waste-water processing techniques in the hospital. To tackle the problem of contrast media in the water system in its entirety, it is necessary for all parties involved to cooperate, from the producer of contrast medium to the consumer of drinking water. This paper aims to make health professionals aware of the opportunity to take the lead now in more conscious decisions regarding use of contrast media and gives an overview of the different perspectives for action.

## Key points

- Contrast media constitute an increasing environmental risk.
- Contrast media contaminate sources for drinking water in many places worldwide.
- Health professionals have an opportunity to reduce the use of contrast media.
- Health professionals can lead the way by initiating measures and necessary conversations.
- Collaboration of stakeholders, health professionals, producers and patients, is essential.

## Background

For radiologists, contrast media are essential for diagnostic and interventional procedures. Iodinated contrast media (ICM) are the most frequently used, particularly in CT scans. The use of ICM is steadily rising due to an increase in CT scanner availability and more medical treatment options that require a CT scan. However, ICMs are increasingly found in surface waters and sources for drinking water and evidence is emerging that ICM breakdown products are toxic [1]. And commonly used drinking water purification techniques are not sufficiently effective in removing ICM, in some cases giving rise to the formation of toxic iodinated disinfection byproducts. Iodinated contrast media pose a problem for drinking water preparation that has not yet been solved. What can we do to limit the environmental impact?

To tackle the problem of contrast media in the water system in its entirety, it is necessary for all parties involved to cooperate, from the producer of contrast medium to the consumer of drinking water. Creating awareness among health professionals is a first step to start active cooperation. Radiologists can do their bit by more conscious use of contrast media.

There are several options for action. Firstly, we can make more conscious decisions regarding use of contrast media. Measures can be implemented to optimize the use of contrast media, to reduce the waste of contrast media and to collect residues of contrast media at the point of application. Secondly, we can implement measures to reduce the amount of contrast media in the sewage water by introducing urine bags or special urine collection and processing techniques in the hospital.

This paper provides radiologists with an overview on the impact of ICM on (drinking) water quality and describes ways to reduce this.

### Iodinated contrast media

Iodinated contrast media are the most commonly used injectables in radiology today. They can be used almost anywhere in the body. Most often, they are used intravenously but can be administered intraarterially, intrathecally, and intraabdominally via an oral or rectal route.

Iodinated contrast media have been in use since the 1950s. The rapid increase in the use of medical imaging during the last few decades has resulted in a substantial increase in the use of contrast media. Over the last 20 years, CT scanner availability has rapidly increased in most countries [2]. In the UK, more than 5 million CT scans are performed per year [3]. In the Netherlands more than 1,9 million [4] and in the USA more than 75 million CT scans are performed every year [5]. So, it involves many millions of CT scans per year worldwide. As a result, many millions of litres (estimated more than 10 million litres) of ICM are used globally every year. This estimate is based on a total of approximately 300 millions CT scans performed per year worldwide [6], of which 40% contrast enhanced CT scans and assuming that 100 ml of contrast medium per CT scan is used.

Modern iodinated contrast media have a high safety profile [7], with a reported excretion of 50% via the urinary tract within about 2 h after intravascular administration in patients with a normal renal function [8–11]. Excretion will take longer [11] in patients with renal function impairment. Due to excretion via the urine, the contrast media enter the aquatic environment and ultimately the drinking water supply system.

### Environmental fate of iodinated contrast media

Since the turn of the century, ICM have increasingly come into focus as environmental contaminants. Simultaneously concern about the impact of these substances on the aquatic environment and the water cycle has grown. The many papers on the environmental fate of ICM were recently reviewed in a review paper published by Sengar and Vijayanandan [1]. It provides an in-depth overview of the vast body of work that has been done on this subject.

The earliest reports of contrast media in the aquatic environment came from Berlin which historically has a short water cycle. Due to its isolated location during the years of Germany’s separation, wastewater treatment plants (WWTP’s) and drinking water productions sites were located in close proximity. The high population density and the associated number of diagnostic tests with ICM within the population led to the discovery of high concentrations of adsorbable organic iodine in hospital wastewater. As analytical development methods for measuring ICM in environmental samples progressed, individual ICM were identified in hospital wastewater, but also in communal wastewater treatment plant influents and effluents.

ICMs have been reported to be present in many surface water systems, rivers and lakes and groundwater systems. Highlighted examples are observations from the Sydney estuary, Australia [12], the Tajo catchment near Madrid [13], the Rhine river and its tributaries [14], the Seine river downstream of Paris [15] and the upper reaches of the Danube near Langenau, Germany [16]. Marine samples from the Baltic Sea (Germany), Northern Adriatic Sea (Italy), Aegean Sea and Dardanelles (Greece & Turkey), San Francisco Bay (USA), Pacific Ocean (USA) and Mediterranean Sea (Israel) were shown to contain ICM [17]. Basically, any body of water that is influenced by communal, wastewater effluents which service urban areas that include diagnostic centers, will most likely show presence of ICM.

The ICMs most frequently reported to be present within the aquatic environment are Iohexol, Iopamidol, Iomeprol, Diatrizoic acid, Iopromide, Ioversol, Iothalamic acid, Iodipamide, Ioxitalamic acid and Iodixanol. Some of these have been part of environmental monitoring programmes for well over 19 years. The annual water quality reports of RIWA-Rijn, the Dutch association of drinking water providers that use the river Rhine as a source, cover data on Diatrizoic acid, Iohexol, Iomeprol, Iopamidol and Iopromide going back to 2002 [18]. In 2020, the ICM load in the Rhine at Lobith (crossing the border between Germany and the Netherlands) ranged between 10.3 and 175 kg/day for individual ICM (Table 1). The total amount of ICM crossing the border from Germany into the Netherlands in 2020 was over 70 tonnes.

**Table 1:** Load of iodinated contrast media in the Rhine at Lobith in 2020

	Mean load	Minimum load	Maximum load	Number of observations
	kg/day	kg/day	kg/day	*n*
Iomeprol	65.3	25.3	175	13
Iopromide	31.4	14.9	85.9	13
Iopamidol	32.5	13.8	47.7	13
Diatrizoic acid	25.9	13.8	73.2	13
Iohexol	38.9	10.3	153	13
Summed daily mean ICM load	194	kg/day		
Summed annual mean ICM load	70.9	ton/year		

Iodinated contrast media (ICM) mean, minimum and maximum daily loads (kg/day), in 2020, in the Rhine at Lobith, the border crossing between Germany and the Netherlands. Also showing the summed mean daily ICM load (kg/day) and summed annual mean ICM load (ton/year)

**Fig. 1:**
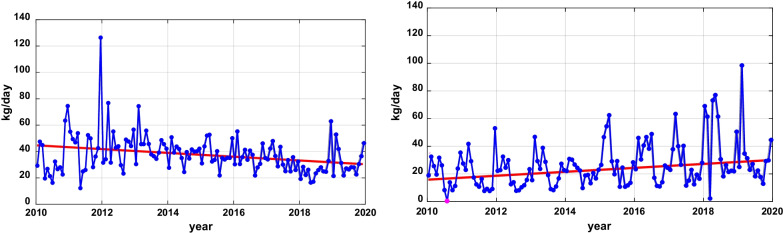
Trends in the daily loads (kg/day) of Diatrizoic acid (left) and Iohexol (right) in the Rhine

The use of these ICM has a long history and, based on their environmental presence, are rightly part of environmental monitoring programmes. Statistical analysis of the trend of the ICM concentrations between the years 2010–2019 at the German-Dutch border crossing (Fig. 1) shows that the load of Diatrizoic acid decreased with 2.7% per year, while Iohexol increased by 3.1% and Iopromide by 2.4% [19]. The load of Iomeprol and Iopamidol did not show a significant trend. The reason for the decrease for Diatrizoic acid can be found in the fact that this oral ICM is in the process of being phased out: oral contrast in the gastrointestinal tract is increasingly substituted by water or barium sulfate-containing contrast media. It has to be noted that it will take a while before new drugs find their way into regulatory water quality monitoring programs. Reduction in environmental concentrations may simply be the result of replacement of an older, monitored compound by a newer, not yet monitored one.

Trends in the daily loads (kg/day) of Diatrizoic acid (left) and Iohexol (right) in the Rhine between 2010 and 2020. The red line denotes a significant trend tested with 95% confidence.

Many of these water bodies also function as a source of drinking water and ICMs pose a threat to drinking water production because of their physico-chemical properties. By design, ICM have a high water-solubility and metabolic stability, which make them difficult to remove during the drinking water purification process. A basic drinking water purification for surface water is ideally based on near natural purification techniques, such as filtration or flocculation to remove suspended matter, slow sand filtration for microbiological degradation of contaminants and activated carbon to remove residual taste, color, and smell. However, these purification steps are incapable of removing ICM entirely.

In situations, where increased levels of contamination have led to an increase in the required amount of purification, additional purification steps have been introduced. Most notably advanced oxidation processes (AOPs), such as ozonation or the use of a combination of UV and hydrogen peroxide have become widespread, depending on the quality of the source water. Although the resulting removal efficacy for ICM is substantially improved, these techniques are known for their side effects, namely the formation of transformation products including from ICMs.

Besides, evidence shows that some ICMs, despite their metabolic stability, can be bio-transformed under environmental conditions that are also relevant to drinking water purification. ICM exhibit biotransformation both under aerobic and anaerobic conditions. This is illustrated by the seasonal fluctuation of Iohexol in Fig. 1: the load of Iohexol fluctuates considerably with seasonal temperature changes. This can be attributed to increased bio-transformation at higher temperatures in the sewerage system and waste water treatment plants as well as in the river itself. On the other hand, Diatrizoic acid does not show this fluctuation and appears to be more resistant to biodegradation. Anaerobic conditions play an important role too when natural drinking water purification is achieved by means of Managed Aquifer Recharge, which may include riverbank infiltration or dune infiltration. Site-specific conditions determine the magnitude of the anoxic-anaerobic transformation processes of ICMs underground, which may vary considerably, and only partial de-iodination may be achieved.

An extensive biotransformation pathway has been described for Iopromide, with both aerobic and anaerobic transformation products. Up to eight transformation processes were reported: reductive deiodination, hydrolysis of anilides, oxidation of primary hydroxyl, oxidation of secondary hydroxyl, cleavage of amide-methylene bond, oxidative decarboxylation, deacetylation and dehydroxylation. All occurred under aerobic conditions, the first two also under anaerobic conditions. However, complete mineralization with formation of pure iodine is not achieved.

Many drinking water suppliers rely on chlorine or chloramine for disinfection or biological stability during transport. The use of chlorination is regulated due to the formation of disinfection by-products (DBP), chloramine being preferred over chlorine due to its reduced reactivity with naturally occurring dissolved organic compounds (DOC) but, in the presence of iodinated contrast media the use of chloramine gives rise to the formation of iodinated disinfection by-products. These were shown to be more genotoxic and cytotoxic than their brominated/chlorinated analogues, with Iodoacetic acid as the most cytotoxic and genotoxic DBP identified [20]. Disinfection by-products (trihalomethanes) show a wide variety of in-vitro and in-vivo effects and toxicity mechanisms [21]. Examples are hepatotoxicity and the formation of hepatic and renal adenomas and adenocarcinomas. The accepted risk limit in drinking water is an excess lifetime risks of 10^–6^ for any potential toxic effect. For the development of kidney cancer, for example, this limit is reached with bromodichloromethane concentrations above 6 μg/L. Chloraminated and chlorinated drinking water from 23 cities in the USA and Canada showed iodo-trihalomethane concentrations that were mostly sub-ng/l levels, yet a maximum concentration of 10.2 μg/l, well above the threshold, was found as well [22]. Whether and how much this threshold concentration is exceeded, will depend on the concentration of iodinated contrast in water supplies used for chlorinated drinking water. Considering the wide variety of disinfection practices dependent on local conditions, it is difficult to provide precise numbers.

Even though ICMs have an intrinsic low toxicity by themselves, it is evident that by-products of their transformation or in wastewater treatment plants, in the environment or drinking water purification may pose a risk for the aquatic environment and ultimately also for our drinking water.

## Measures to reduce the use of contrast media

### Individualizing the volume of intravenous iodinated contrast medium in contrast-enhanced CT

More conscious use of intravenous contrast media provides a means to mitigate the problem at its source. In the 1980s and 1990s, the same volume of contrast medium per patient has been used, independent of the weight of the patient and only dependent on the scanning protocol. Since then, much progress has been made in personalizing contrast volume to achieve the desired image quality. Weight categories were introduced to adapt contrast volume per category. Nowadays, individual adjustment of contrast volume has been introduced by a growing number of institutions, determined the clinical question and personalized to body weight, lean body mass or body surface area [23]. For each CT protocol, volume and injection rates are calculated individually. At our institution, a contrast calculator based on patient weight is routinely applied by our radiographers.

### Reducing the amount of contrast media for contrast-enhanced CT

Three techniques hold potential for being able to reduce the total amount contrast needed for adequate contrast-enhanced CT scanning: low-kV techniques, dual energy scanning with reconstruction of low-keV images, and the contrast boost technique for CT angiography, based on CT subtraction. At present, however, these techniques are mainly used if there is a *clinical* indication to minimize the amount of contrast material, mainly in patient with reduced renal function. No major efforts have been made to use these techniques to generally reduce the amount of contrast material.

At low x-ray energies, the x-ray absorption of iodine increases rapidly, which can be used to get better enhancement for the same amount of ICM injected or similar enhancement with less ICM. *Low kV scanning* has been initially used for CTA (CT angiography) to increase arterial enhancement [24] but is nowadays used to optimize any type of contrast-enhanced CT. It fails if the x-ray absorption is too large, like in abdominal exams of obese patients, because then the higher enhancement is counteracted by a much increased image noise.

*Dual energy* techniques allow for calculating low keV images [25]. Since keV can be chosen lower than the equivalent for low-kV scanning, even higher iodine enhancement can be gained for the same amount of contrast medium. The technique requires scanners with dedicated hardware and best works in conjunction with elaborate noise-reduction algorithms. It is mainly used to reduce contrast dose in patients with reduced renal function [26].

The *contrast boost* technique is a new way to boost contrast enhancement but is at present limited to a specific CT vendor (^SURE^Subtraction, Canon Medical Systems). By subtracting a pre-contrast image from the contrast-enhanced image, an enhancement map is created that can then be added (with a weighing factor) to the contrast-enhanced image to boost contrast. The technique requires an additional (low-dose) pre-contrast image and software for non-rigid image registration to avoid misregistration artefacts. In principle, the technique is superior in terms of CNR (contrast to noise ratio) to the low-keV technique with dual energy scanning but at present, no good noise reduction algorithms are available to fully exploit these advantages. Main indication at present is image quality improvement for CTA; application for reducing the amount of contrast media have not yet been tried. Since a (low-dose) pre-contrast scan is added, the additional radiation dose becomes an issue. In cases where the amount of contrast medium needs to be minimized for clinical indications, such as reduced kidney function, the benefits outweigh the radiation risk; for reducing contrast material in waste water, it is not suited yet. It could become applicable if both, the pre-and post-contrast image, are used for advanced noise suppression, thus not using also the radiation dose of the additional pre-contrast scan for image formation.

### Replacing oral contrast media by water for abdominal CT scan

In the past positive oral contrast was used in the majority of abdominal CT scans. Oral contrast plays an important role for bowel distension and to improve the evaluation of the gastrointestinal mucosa. However, the focus for most scans is not on the gastrointestinal mucosa and/or bowel distension, but on the evaluation of visceral or metastatic disease. Research shows that oral water can replace positive oral contrast in the majority of abdominal CT scans [27, 28]. Selected clinical questions, such as pelvic abscess or fistula to an intestine, still require individual oral contrast preparations.

## Measures to reduce the waste of contrast media

### Multi-patient injection system

Contrast material that has been prepared for patient injection but has not been used is discarded and thus, wasted. In addition to unnecessary costs, it leads to an increased environmental burden.

Multi-patient injection systems allow for using flexible bottle sizes ranging from 50 to 500 ml. This makes it possible to individualize the amount of contrast material injected without increasing contrast waste.

The system optimally operates by starting the day with a bottle size of 500 ml and then adjusting bottle sizes to the expected aggregated use toward the upcoming scan hours at the end of the day [29], taking into account the maximum use time once the bottle stopper has been pierced. This time varies from 10 to 24 h, depending on manufacturer. If 24 h are allowed, 500 ml bottles can be used throughout the week and the amount of wasted (unused) contrast material is minimized.

### Saline flush technique

The saline flush, also known as a saline chaser, is a secondary injection following the administration of contrast medium via a power injector [30]. The primary purpose of the saline chaser is to ‘push’ the otherwise unused contrast medium in connecting tubing and the peripheral vessels toward the heart to make sure all contrast material contributes to the desired enhancement.

## Measures to collect residues of contrast media

### Collection of residues of contrast media

Collecting residues of contrast media separately and disposing them via specific hospital waste channels prevents contrast entering the sewerage system. At our institution, a special container is present in each CT suite and the angio suites to collect contrast media residues. These containers are disposed of via the specific hospital waste channels and are destroyed in an incinerator. This destroys the chemical composition of the contrast material and produces molecular iodine (I_2_) and iodine salts, which are also naturally present in the environment [31]. In the past, these residues were simply disposed of by pouring the contrast material down the sink.

### Iodine recycling services

Iodine recycling is a novel opportunity. One producer of contrast media (GE Healthcare) offers a service to collect and recycle uncontaminated iodinated contrast media leftovers. Special containers are delivered to the radiology departments in hospitals and are collected by the producer. The iodine is extracted from the substance and used for production of new contrast media [32].

## Measures to reduce the amount of contrast media in sewage water

Injected contrast material is excreted via the urine and, to a much lesser amount, via the liver and biliary system. To reduce the amount ending up in the environment, excreted urine after contrast administration can be collected separately or hospital waste water can be treated before entering the sewage system.

### Urine bags after contrast administration in outpatients

Disposable urine bags contain an absorbent material that fixes the urine and are sealable. The patients use them at home during the first four urination sessions after intravenous contrast administration. The bags are disposed of via the household waste system*.* If this household waste is further processed in an incinerator, the contrast material is reduced to naturally occurring iodine and iodine salts [31] which are not known to have a negative environmental impact. Urine bags and ICMs which end up in landfills will equally have no negative environmental impact assuming the landfill site is properly managed. Physical barriers such as clay or synthetic liners and/or collecting and treating landfill leachate will prevent contamination of the surrounding groundwater. If such measures are not present, other landfill related contaminants will pose a much larger threat to the environment than ICMs.

In the Netherlands, a pilot study was carried out in six hospitals. Outpatients received four urine bags after a contrast enhanced CT. Most patients were willing to participate and indicated that they would like to contribute to a better environment. Providing clear instructions for use was important. Most radiographers were able to do this with no or minimum extra time compared to a regular workflow. Involvement of radiographers and staff has been shown to be crucial during introduction of these measures [33].

In Germany, a pilot project with urine bags was performed in 2200 patients. The patients received a package with four urine bags and instructions for use. The vast majority of patients (87%) indicated that they had used the urine bags. Measurements taken in wastewater showed a 45% lower concentration of contrast medium compared to the period prior to the pilot study [34].

The ecological footprint of the destruction of urine bags is limited. Research shows that the environmental impact when contrast media are processed in urine bags through waste incinerators is smaller than when the contrast media are processed in sewage treatment plants [31].

Municipal waste usually contains very small quantities of iodine compounds. Iodine emissions are, therefore, of minor importance to municipal waste incineration plants [35]. Considering the amount of iodine that would be added per patient in comparison with the total amount of municipal waste of the entire population, it is reasonable to expect no negative environmental impact. Current incineration protocols already deal with the presence of halogens (chlorine and bromine) in municipal waste and suffice for the safe disposal of iodinated substances.

### Separate collection of urine after contrast administration in hospitalized patients

Separate collection of urine after contrast enhanced CT or interventional procedures in the hospital can be implemented by installing toilets for separate collection of urine with waterless urinal technology. These toilets must be connected to a special storage container.

### On site medical waste treatment

A comprehensive approach on pharmaceutical residues in hospital wastewater might be found in dedicated on site medical waste treatment. An example of this is the pharmafilter concept which aims at treating both solid and liquid hospital waste in fermenter system near the hospital. The system produces biogas and removes a wide range of pharmaceuticals, including antibiotics from hospital wastewater. A pilot study in the Netherlands showed a complete removal of ICM from hospital wastewater [36]. Implementation of the pharmafilter concept may however prove to be more feasible in newly designed hospitals but would, as an added benefit, remove many pharmaceuticals with a known environmental impact.

## Environmental sustainability

### Awareness

Creating awareness is essential. As healthcare professionals, we are trained to provide primary patient care. For a radiologist, image quality is paramount: the use of contrast media often is necessary for answering the clinical question. However, many radiologists and radiographers have no knowledge about the impact of contrast media on water quality. As healthcare professionals, we also have a responsibility for the environment. When using contrast media, we should be aware of their environmental impact. Awareness of the environmental impact has then to be followed by active participation in prevention programs to reduce the amount of contrast material residues that reach our water supplies.

### Cooperation

Creating cooperation of all involved parties is essential for a comprehensive approach. To achieve success, a joint approach is needed with sufficient support from relevant authorities. Ideally, producers, pharmacists, radiologists, radiographers, patients, hospital representatives, drinking water supplier representatives, regional water authorities, and health insurers need to collaborate. One example is the government program “Reducing pharmaceutical residues in water: a chain approach,” which has recently started in the Netherlands [37] and involved the majority of these parties.

## Future directions

Sustainability is receiving increasing attention within the healthcare sector. Both hospitals and pharmaceutical companies are trying to reduce their ecological footprint and improve their sustainability. Among physicians and pharmacists, there is a growing focus on optimizing medication use [38] and on reducing the waste of medication [39]. Within radiology, there is still a great deal of potential for savings through the introduction of the measures mentioned.

The demand for raw materials in the world is increasing further as the wealth levels of citizens in emerging economies are on the rise. At present, most iodine used in contrast material production comes from Chile and Japan, which makes the industry vulnerable to production shortages and ultimately limited (easily exploitable) natural iodine resources. The expected scarcity of raw materials will force more and more companies to sustainable resource management. In the long term, it is desirable to be able to recycle contrast media from the urine of patients. This saves raw materials and contributes to a circular process.

A company (Bayer AG (Bergkamen site, Germany)) has implemented recycling procedures to reduce the amount Iopromide reaching their wastewater treatment plant. Organically bound iodine is recovered to recycle the iodine, thereby reducing both their resource demand and their environmental impact on the Lippe River, a tributary of the Rhine [40].

In 2021, BIPSO (Bracco Imaging Pharmaceutical Sterile Operations) GmbH (Singen, Germany) started collecting and concentrating "first rinse wastewater," which is generated within their sterile production line. By implementing reverse-osmosis technology, a reduction of the emission of ICM and Gd-based contrast media is achieved of over 90%. Besides reducing the amount of contrast media in wastewater significantly, iodine is recycled from the concentrated waste [41].

Considering the growing knowledge on how ICMs and their breakdown products negatively impact the environment, this knowledge also provides a unique opportunity. Taking into account what makes ICMs bio-degradable and how to avoid toxic by-products, this knowledge could give direction to future product development. Using "benign by design" development principles [42], new ICMs may have a lesser environmental impact and mitigation measures proposed in this paper might be scaled down over time.

Medical imaging techniques, especially those using contrast material, have been steadily rising over the past decade and they continue to do so now. Many modern treatments, especially in oncology, require more follow-up, usually using contrast-enhanced CT. Better access to imaging in developing countries further contribute to increase demand in contrast material worldwide. Developing techniques to recycle the raw materials needed for production of contrast materials may become an important topic for the sustainability of healthcare worldwide.

## Conclusions

Iodinated contrast media are used in large quantities, particularly in CT scans. Due to excretion through the urine, the contrast media enter the water supply system. In many places around the world, contrast media have been shown to be present in drinking water. If we do not change this, we will be faced with lifelong exposure to contrast media and their breakdown products, simply through drinking water. Consumers expect clean and wholesome drinking water. As health professionals, we can pick up the gauntlet to work on awareness and cooperation to tackle the problem of this contamination of our drinking water.

We have worked under the assumption that contrast media are stable, inert, and non-degradable under environmental conditions, but the evidence shows that is not true. Increasingly, evidence is being presented that unintended effects arise due to breakdown products, either through biotic or abiotic processes.

There are several options for action available, which we can work from. Firstly, we can make more conscious decisions regarding use of contrast media. Measures can be implemented straight away to optimize the use of contrast media, to reduce the waste of contrast media and to collect residues of contrast media at the point of application. Secondly, we can implement measures to reduce the amount of contrast media in the sewage water by introducing urine bags or special urine collection and treatment techniques in hospitals.

To speed up the process, it is important to create awareness among radiologists, radiographers, the pharmaceutical industry and among patients. Pilot studies show that patients appear to be happy to make an effort themselves for a clean environment and ultimately their drinking water. Patient education plays an important role in this respect: we can, for example, use patient information films in waiting rooms and digital patient information leaflets via the hospital's digital patient portal. But also, social media involvement will help get the word out that iodinated contrast media do not need to threaten our environment and our drinking water because something can be done, and it can be done by the consumers themselves.

To tackle the problem of ICM in the water system in its entirety, it is necessary for all parties involved to cooperate, from the producer of contrast medium to the consumer of drinking water. If everyone involved in this chain does their bit, the environmental exposure and subsequent downstream effects can be greatly reduced and ultimately mitigated.

## Data Availability

Not applicable.

## Abbreviations

AOPs: Advanced oxidation processes
CNR: Contrast-to-noise ratio
CTA: CT angiography
DBP: Disinfection by-products
DOC: Dissolved organic compounds
ICM: Iodinated contrast media
RIWA-Rijn: The Dutch association of drinking water providers dependent on the river Rhine
WWTP’s: Wastewater treatment plants
